# Entanglement goes classically high-dimensional

**DOI:** 10.1038/s41377-021-00521-w

**Published:** 2021-04-15

**Authors:** Qiwen Zhan

**Affiliations:** School of Optical-Electrical and Computer Engineering, University of Shanghai for Science and Technology, Shanghai, China

**Keywords:** Single photons and quantum effects, Solid-state lasers

## Abstract

Laser beams from a customarily designed resonator can produce vectorial structured light fields as classical analogs to high-dimensional multipartite quantum entangled states.

Quantum mechanics is one of the greatest achievements of the twentieth century and has fundamentally changed the way we think about the physical universe. However, it is also one of the most mysterious scientific theories. At the core of this mystery is the so-called quantum entanglement. Measurement of this superposition of quantum states shows that a strong nonclassical correlation can exist between two distantly separated quantum systems, leading to the so-called quantum “spooky action at a distance”^1^. This represents one of the most striking outcomes of quantum mechanics and serves as the most fundamental quantum mechanical resource, playing an important role in many quantum computing and quantum information applications.

Entanglement used to be discussed strictly in the quantum context. One of its key properties is nonlocality, meaning that the measurement of one quantum system appears to affect the state of an entangled quantum system a certain distance away, seemingly contradicting special relativity. This correlation is tested by Bell’s measure^2^ to reject the local “hidden variable” argued in the Einstein–Podolsky–Rosen (EPR) paradox^3^. Quantum entanglement serves as the foundation of quantum computing and quantum information. However, drawbacks in the realization and application of these quantum entangled states include the requirement of coincident “clicking” detection due to the low signal level and susceptibility to degradation due to the environment. Recently, there has been much interest in constructing entanglement states using classical optical fields in the hope of retaining some of the features that are analogs to quantum entanglement while avoiding the drawbacks and pushing the quantum-classical boundary^4–8^. Entanglement prepared with classical electromagnetic fields is consequently referred to as classical entanglement.

There have been debates on the nomenclature regarding whether we should associate entanglement with classical fields^9–11^. Setting aside the physical quantity involved, the consensus is that mathematically, they share the same “nonseparable” characteristic. However, unlike quantum entangled states, which often deal with the entangled degrees of freedom (DoFs) of two different particles (intersystem), classical entanglement deals with different DoFs within the same optical field (intrasystem). Hence, nonlocality is not an issue, and their correlation should be purely classical in nature. Nevertheless, it has been shown that Bell’s measure can still be defined in the context of optical coherence^12^. Opposed to serving as an indicator for the violation of local realism, Bell’s measure for classically entangled states serves as a bound for the degree of coherence that is accessible to a system^12^. Frameworks developed in quantum computing, quantum information, etc., can still be applied to classically entangled fields and find applications in, e.g., imaging, computing, information processing and metrology, despite the lack of a quantum nonlocality feature^13^.

The most popular approach to creating classical entanglement is to use the DoFs in spatial modes and the angular momentum, including the spin angular momentum (SAM, or polarization) and orbital angular momentum (OAM). One example of this classical entanglement with spatially variant polarization is illustrated in Fig. 1^14^. In this case, the spatial mode and the polarization bases cannot be separated mathematically. In other words, they cannot be expressed as a product of the spatial modes with the polarization bases. The classically entangled states prepared in this way have been limited to two DoFs and two dimensions as classical analogs to two-photon qubits. The ability to access more DoFs for high-dimensional state spaces would be very beneficial, offering access to many exciting applications.

**Fig. 1:**
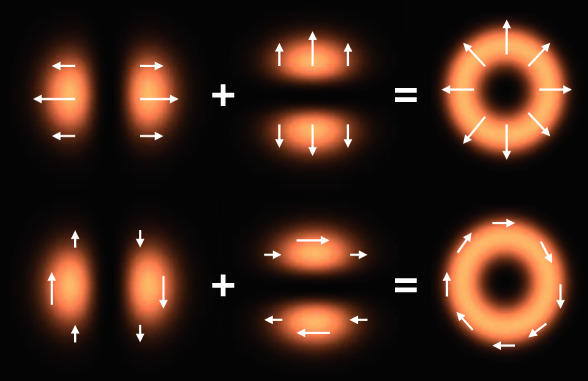
Formation of radially and azimuthally polarized beams using the linear superposition of orthogonally polarized HG modes. The intensity distribution and the polarization pattern of these kinds of beams are considered to be nonseparable or entangled

Marked advances have been made in the generation of vectorial optical fields. A variety of approaches to creating complex vectorial fields, including intracavity and extracavity methods, are now available^14–16^. The DoFs added by these vectorial optical fields enable the generation and manipulation of higher-dimensional classical entanglements. In the work by Forbes et al., eight-dimensional classical entanglement states are demonstrated^17^. Using a cavity design and ray-wave duality to produce a paraxial coherent spatial wavepacket, they exploit three DoFs (position, direction of propagation, and polarization) in the state design. Control over the position and direction of propagation is realized with the cavity mirror arrangement. Control over the polarization is realized with a c-cut crystal in the cavity and angular-dependent birefringence at a nonnormal incident angle. Each of the DoFs could take two possible states. Hence, the system provides 8-dimensional classical entanglement of three partitions. The authors demonstrate the capability of their approach through the generation of a complete set of classical Greenberger–Horne–Zeilinger (GHZ) states analogous to high-dimensional multipartite quantum entangled states^18^. To test the fidelity of the generated classical GHZ states, a tomography technique based on the Bell projection is developed and implemented. These GHZ states can be used for quantum simulation in a classical optics framework.

The work also provides an expandable platform for scaling up to even higher-dimensional states. For example, the OAM DoF can be straightforwardly incorporated to form four-partite entangled states. In addition to these DoFs that are already well known, recent advances made in vectorial optical fields offer nontraditional DoFs that could further increase the dimensionality and number of partitions involved. For example, it has been found that optical fields can carry transverse SAM that is orthogonal to the propagation direction using carefully designed vectorial optical fields^19^. Recently, ultrafast spatiotemporal wavepackets carrying transverse OAM that is perpendicular to the propagation direction have been demonstrated^20^. Continuous development in vectorial complex fields and spatiotemporally structured light will continue to supply new tool sets that can be exploited in classical entanglement studies and further push the quantum-classical boundary.
